# Supplementary material for: Dynamical patterns and nonreciprocal effective interactions in an active-passive mixture through exact hydrodynamic analysis
Source: Nat Commun. 2025 Jul 1;16:6017. doi: 10.1038/s41467-025-60518-6 (PMC12217814; doi:10.1038/s41467-025-60518-6)
Supplement: Supplementary file 1 — Supplementary Information [file 41467_2025_60518_MOESM1_ESM.pdf]

Supplementary Information for: "Dynamical patterns and nonreciprocal effective interactions in an active-passive mixture through exact hydrodynamic analysis"

James Mason,[1] Robert L. Jack,[1,2] and Maria Bruna[1,3]
[1]DAMTP, Centre for Mathematical Sciences, University of Cambridge, Wilberforce Road, Cambridge CB3 0WA, UK
[2]Yusuf Hamied Department of Chemistry, University of Cambridge, Lensfield Road, Cambridge CB2 1EW, UK
[3]Mathematical Institute, University of Oxford, Oxford OX2 6GG, UK

A. HYDRODYNAMIC LIMIT

The hydrodynamic system of PDEs (1) describes the local density of each type of particle as the lattice spacing $h \to 0$. For ease of reference, we rewrite (1) below in terms of $\rho = \rho_+ + \rho_- + \rho_0$, $\rho_a = \rho_+ + \rho_-$ and $m = \rho_+ - \rho_-$:

$$\partial_t \rho = \nabla \cdot \nabla \rho - \mathrm{Pe}\partial_x \left[(1-\rho)m\right], \tag{S1a}$$

$$\partial_t \rho_a = \nabla \cdot \left[d_s(\rho)\nabla \rho_a + \rho_a \mathcal{D}(\rho)\nabla \rho\right] - \mathrm{Pe}\partial_x \left[\rho_a s(\rho) m + d_s(\rho) m\right], \tag{S1b}$$

$$\partial_t m = \nabla \cdot \left[d_s(\rho)\nabla m + m\mathcal{D}(\rho)\nabla \rho\right] - \mathrm{Pe}\partial_x \left[s(\rho)m^2 + d_s(\rho)\rho_a\right] - 2m. \tag{S1c}$$

To obtain these equations from the particle-based dynamics of the APLG, we define $\eta_\sigma(\mathbf{x}, t)$ to be 1 if there is a σ-particle at position $\mathbf{x}$ and 0 otherwise. The local density is formally defined as the mean number of particles in a mesoscopic box of radius r around $\mathbf{x}$,

$$\hat{\rho}_\sigma(\mathbf{x}, t) \approx \frac{1}{(2r/h+1)^2} \sum_{\|\mathbf{x}-\mathbf{y}\|_\infty < r} \eta_\sigma(\mathbf{y}, t), \tag{S2}$$

for $1 \gg r \gg h$. Recalling that the lattice is embedded in a physical domain of size $\ell_x \times \ell_y$ and that the total numbers of active (and passive) particles per site are ϕ_a (and ϕ_p), the relevant limit is $\ell_y \to 0$ at fixed $\ell_x, \ell_y, \phi_a, \phi_p$. The hydrodynamic limit exists if that the random variables $\hat{\rho}_\sigma$ converge (in probability) to deterministic densities ρ_σ, which are solutions to the hydrodynamic PDE system (S1).

This is proven in the APLG by generalizing the work of Erignoux [1]. That work considered a system of pure active particles with continuously varying orientations $(\cos\theta, \sin\theta)$ with $\theta \in [0, 2\pi)$ instead of only $\theta = \{0, \pi\}$ used here. In both cases, the proof of the convergence $\hat{\rho}_\sigma \to \rho_\sigma$ is technically challenging because the models are of nongradient type in the sense of [2]. It is worth emphasizing that this classification is separate from whether the model can be derived as a gradient flow of an equilibrium free energy. Indeed, the hydrodynamic limit of a mixture of two species undergoing a symmetric simple exclusion process is a gradient flow, but the underlying microscopic model is of nongradient type [3]. Being of nongradient type means instead that the current $j_{\mathbf{x},\mathbf{x}+h\mathbf{e}_i}$ from site $\mathbf{x}$ to $\mathbf{x} + h\mathbf{e}_i$, cannot be written as the discrete difference of a local function g

$$j_{\mathbf{x},\mathbf{x}+h\mathbf{e}_i} \neq g_{\mathbf{x}+h\mathbf{e}_i}(\eta) - g_{\mathbf{x}}(\eta). \tag{S3}$$

The original proof in [1] is extremely technical and long; for an abridged version, we refer the reader to [4, Sec. 5].

The nongradient method [3] involves projecting the current onto a space of discrete differences and proving that it can be replaced by its local average in the hydrodynamic limit, e.g., the instantaneous current of σ-particles going between neighboring sites $\mathbf{x}$ and $\mathbf{x} + h\mathbf{e}_1$ can be approximated as

$$j^\sigma_{\mathbf{x},\mathbf{x}+h\mathbf{e}_1} \simeq d_s(\rho)[\eta_\sigma(\mathbf{x}+h\mathbf{e}_1) - \eta_\sigma(\mathbf{x})] + \mathcal{D}(\rho)[\eta(\mathbf{x}+h\mathbf{e}_1) - \eta(\mathbf{x})] + \mathrm{Pe}\left[\sigma d_s(\rho)\eta_\sigma(\mathbf{x}) + s(\rho)m\eta_\sigma(\mathbf{x})\right], \tag{S4}$$

see [4, §5(c)] for details. It is the symmetric part of the dynamics (shared by the active and passive particles and leading to the first two terms in the right-hand side of (S4)) of the APLG that makes the model of nongradient type. As a result, adding a different type of particles (with identical symmetric jump rates) does not bring new challenges. As such, while [1, 4] cannot be used verbatim, the proof of the APLG hydrodynamic limit is a straightforward generalization of those works.

Another signature of the model being of nongradient type is that its hydrodynamic limit contains transport coefficients whose dependence on the density is not known, albeit they are characterized by a variational formula over an infinite-dimensional space [5]. This is the case for the self-diffusion coefficient $d_s(\rho)$ in Eq. (S1). In our analysis, we use the approximation (3) of $d_s(\rho)$ obtained in [6], exploiting a rigorous recursive approach proposed in [7]. The

idea equates to performing a Taylor expansion of $d_s(\rho)$ around $\rho = 0, 1$ up to the first order and combining the two linear approximations in the minimal cubic polynomial. At the level of the self-diffusion coefficient, the mean-field approximation $\langle \eta_\sigma(\mathbf{x}, t)\eta_\sigma(\hat{\mathbf{x}}, t)\rangle \approx \rho_\sigma(\mathbf{x}, t)\rho_\sigma(\hat{\mathbf{x}}, t)$ would result in $d_s(\rho) \equiv 1 - \rho$ in (3) and, in turn, $\mathcal{D}(\rho) \equiv 1$ and $s(\rho) \equiv 0$ in (2).

1. Choices of scaling limit

As discussed in the main text, the microscopic rates of the APLG exhibit a nontrivial dependence on h. For instance, the hopping rate scales as $O(h^{-2})$, while the orientation flip rate is $O(1)$. In the hydrodynamic limit $h \to 0$, the rapid hopping effectively mixes the system, a crucial ingredient in the proof of (S4). Consequently, the densities ρ_+ and ρ_- emerge as hydrodynamic (slow) fields. This behavior contrasts with NRCH-like frameworks for active-passive mixtures [8–10], where the hydrodynamic fields would typically be the total active density ρ_a (summed over orientations) and the passive density ρ_0, while the magnetization m would be a fast (nonhydrodynamic) field.

Physically, the magnetization field is "fast" if the timescale for orientational relaxation is comparable with the time a particle takes to diffuse a distance comparable with its own size. The rigorous mathematical analysis of [4] is not possible in this case: it relies on a separation of time scales between hopping and orientational relaxation. In this sense, the time-scale separation of the APLG is an idealized modeling assumption, which is widespread in interacting particle systems [1, 11–13]. Nevertheless, the APLG supports complex behavior reminiscent of other active-passive mixtures without any such timescale separation [12, 14–16]. That is, the simplified APLG model still captures the emergent behavior of its more complex counterparts.

We also comment on the thermodynamic limit, which is distinct from the hydrodynamic limit. The thermodynamic limit takes a large system $\ell_x, \ell_y \to \infty$ with all other parameters (including h) held fixed. Such scenarios are commonly studied in physics; see [17] for a recent purely active example that shares strong similarities with the APLG. The analysis of [17] does not involve any time scale separation between orientational relaxation and local mixing, which means that fluctuations affect the observed behavior, leading (for example) to bubbly phase separation. Such effects also lead generically to critical points with nontrivial (non-Gaussian) scaling exponents. Neither of these effects can be derived from noise-free hydrodynamic equations like (1), which describe the APLG. The reason is that the hydrodynamic scaling limit of the APLG is constructed in a way that suppresses fluctuations. This allows for exact analysis but suppresses fluctuation-dominated effects like non-Gaussian critical exponents.

B. NONRECIPROCITY

This section explains how the hydrodynamic system (S1) corresponds to a system with nonreciprocal effective interactions. We briefly review the origin of the "nonreciprocal" terminology in violations of Newton's third law in finite systems of interacting particles. After that, we discuss nonreciprocal effective interactions for interacting density fields.

1. Ordinary differential equations

We analyse a finite system of N interacting particles, focusing on the behaviour near a fixed point (see [18] for an analysis of bifurcations and phase transitions). The particles have co-ordinates $x_1, x_2, \ldots, x_N$ and follow deterministic overdamped dynamics. Write $\mathbf{x} = (x_1, x_2, \ldots)$ and define an energy $E = E(\mathbf{x})$. We assume that E is translation-invariant, that is

$$\sum_i \frac{\partial E}{\partial x_i} = 0 \,. \tag{S5}$$

This assumption simplifies the analysis by ensuring that all forces can be attributed to interparticle interactions, whose behaviour is consistent with Newton's third law, see below. (There are no "body forces" or external forces.) More general cases can also be analysed in a similar way.

The overdamped dynamics for a reciprocal system is

$$\dot{\mathbf{x}} = -\Gamma^{-1}\nabla E, \tag{S6}$$

where Γ is the friction matrix, which is real, symmetric, and positive definite. The energy E is nonincreasing under the dynamics (S6). Assume that $\mathbf{x} = 0$ is a fixed point [$\nabla E(0) = 0$] and linearise (S6):

$$\dot{\mathbf{x}} = B\mathbf{x}, \qquad B = -\Gamma_0^{-1} H_0, \tag{S7}$$

where H_0 is the Hessian of E at $\mathbf{x} = 0$ and $\Gamma_0 = \Gamma(0)$. Stability depends on the eigenvalues of B. Using that Γ_0 is real symmetric positive definite and similarity-transforming with $\Gamma_0^{1/2}$, the eigenvalues of B are those of the real symmetric matrix $-\Gamma_0^{-1/2} H_0 \Gamma_0^{-1/2}$, hence they are always real. If the eigenvalues of H_0 are positive (minimum of E), then the eigenvalues of B are negative (stable fixed point, as expected). The translation invariance (S5) means that H_0 has a zero eigenvalue, but this may always by removed by defining positions relative to the center of mass.

We have from (S5) that $\sum_i (H_0)_{ij} = 0$ which allows (S7) to be written in components as

$$(\Gamma_0 \dot{\mathbf{x}})_i = \sum_{j(\neq i)} F^{\mathrm{p}}_{ij}, \qquad F^{\mathrm{p}}_{ij} = (H_0)_{ij}(x_i - x_j)\ , \tag{S8}$$

where we identify F^{p}_{ij} as the (pairwise) force on particle i from particle j. The Hessian is symmetric, so this recovers Newton's third law as

$$F^{\mathrm{p}}_{ij} = -F^{\mathrm{p}}_{ji} \tag{S9}$$

(Note: we did not assume that the energy is a sum of pairwise additive contributions, this formula giving F^{p}_{ij} in terms of the Hessian is always valid close to the fixed point.)

To generalise (S6) to non-reciprocal systems, we write

$$\dot{\mathbf{x}} = \Gamma^{-1}\mathbf{F}, \tag{S10}$$

where $\mathbf{F}$ is a force, which is not generically the gradient of any energy E. We continue to assume that $\mathbf{x} = 0$ is a fixed point and translation invariance (now in the form $\sum_j (\partial F_i/\partial x_j) = 0$ for all i). Linearising about the fixed point gives the analog of (S8)

$$(\Gamma_0 \dot{\mathbf{x}})_i = \sum_{j(\neq i)} F^{\mathrm{p}}_{ij}, \qquad F^{\mathrm{p}}_{ij} = \frac{\partial F_i}{\partial x_j}(x_j - x_i) \tag{S11}$$

where again F^{p}_{ij} is the force on particle i from particle j. If $\mathbf{F} = -\nabla E$ then we recover the previous case, including Newton's third law (S9), but this is violated if $\frac{\partial F_i}{\partial x_j} \neq \frac{\partial F_j}{\partial x_i}$. Striking examples occur when particle i is attracted towards particle j, but particle j is repelled from particle i, leading to predator-prey-type dynamics [19, Chapter 3].

It is also useful to write the linearised system (S11) as

$$\dot{\mathbf{x}} = B_{\mathrm{NR}}\mathbf{x}, \qquad B_{\mathrm{NR}} = \Gamma_0^{-1} J, \qquad J_{ij} = \frac{\partial F_i}{\partial x_j} \tag{S12}$$

analogous to (S7). The matrix B_{NR} may have complex eigenvalues (because J is not symmetric in general). We note that for any given equation of motion (S10), it may not be obvious whether it can be factorised as (S6). However, if linear stability yields complex eigenvalues, then such a factorisation is not possible, and the system must be nonreciprocal.

Example 1 (Non-reciprocal ODE system)**.** Consider three particles with positions x_1, x_2, x_3 moving in the interval $[0, 2\pi)$ with periodic boundaries. We take as the equation of motion

$$\begin{pmatrix} \dot{x}_1 \\ \dot{x}_2 \\ \dot{x}_3 \end{pmatrix} = \begin{pmatrix} (1+a)\sin(x_2 - x_1) + (1-a)\sin(x_3 - x_1) \\ (1+a)\sin(x_3 - x_2) + (1-a)\sin(x_1 - x_2) \\ (1+a)\sin(x_1 - x_3) + (1-a)\sin(x_2 - x_3) \end{pmatrix} \tag{S13}$$

where the friction matrix is $\Gamma_0 = \mathbf{1}$. The fact that forces only depend on particle separations ensures translation invariance, and allows individual terms to be easily identified as interparticle forces.

For $a = 0$ this is a reciprocal system with $E = -[\cos(x_1 - x_2) + \cos(x_1 - x_3) + \cos(x_2 - x_3)]$: all forces are attractive and setting $x_1 = x_2 = x_3$ gives a fixed point. For $0 < |a| \leq 1$ some attractive forces are weakened while others are strengthened, which breaks reciprocity; for $|a| > 1$ some forces become repulsive so that (for example) particle 1 may be attracted to particle 2 while particle 2 is repelled from particle 1. Nevertheless, the fixed point remains the same.

Linearising about this point we arrive at the situation described in (S11), with

$$F_{12}^{\mathrm{p}} = (1+a)(x_2 - x_1), \qquad F_{21}^{\mathrm{p}} = (1-a)(x_1 - x_2), \tag{S14}$$

being the forces between particles 1 and 2. Clearly $F_{12}^{\mathrm{p}} \neq -F_{21}^{\mathrm{p}}$ and Newton's third law is violated, unless $a = 0$. The situation is similar for forces between other pairs of particles. Writing the linearised equation of motion as in (S12) gives

$$B_{\mathrm{NR}} = \begin{pmatrix} -2 & 1+a & 1-a \\ 1-a & -2 & 1+a \\ 1+a & 1-a & -2 \end{pmatrix} \tag{S15}$$

whose eigenvalues are $0, -3 \pm ia\sqrt{3}$. The fixed point is (marginally) stable in the reciprocal case and this is unchanged by non-reciprocity. However, any non-zero a yields complex eigenvalues, indicating oscillatory decay to the fixed point. The existence of oscillations for all $a \neq 0$ is not generic: it occurs because the underlying reciprocal system has degenerate eigenvalues. (It is straightforward to construct similar systems where the eigenvalues remain real for some finite range of a.) However, complex eigenvalues are not compatible with reciprocal forces.

2. Cross-diffusion systems

Now consider two species with densities ρ_1 and ρ_2 that vary in space. A generic reciprocal PDE system (for example describing relaxation towards thermal equilibrium) analogous to (S6) is:

$$\partial_t \begin{pmatrix} \rho_1 \\ \rho_2 \end{pmatrix} = \nabla \cdot \left[M \nabla \begin{pmatrix} \delta E/\delta \rho_1 \\ \delta E/\delta \rho_2 \end{pmatrix} \right], \tag{S16}$$

where the mobility matrix M is real symmetric positive definite (in general it may depend on ρ_1, ρ_2), and E is the free energy. Assuming for simplicity that $E = \int \varepsilon(\rho_1, \rho_2) \mathrm{d}x$ so that $\delta E/\delta \rho_i = \partial \varepsilon / \partial \rho_i$ for $i = 1, 2$, (S16) reduces to the cross-diffusion problem

$$\partial_t \begin{pmatrix} \rho_1 \\ \rho_2 \end{pmatrix} = \nabla \cdot \left[M H \begin{pmatrix} \nabla \rho_1 \\ \nabla \rho_2 \end{pmatrix} \right], \tag{S17}$$

where H is the Hessian matrix of ε. The steady states ρ_i^∞ of (S17) are the minimisers of E and satisfy $\partial \varepsilon / \partial \rho_i$ constant. For M linear, this is the set-up of Onsager's reciprocity principle [20], in which the macroscopic symmetry of a system close to equilibrium arises from its microscopic time reversibility, see also [21].

Linearising (S17) about ρ_i^∞ and taking $\rho_i = \rho_i^\infty + e^{-iqx} f_i(t)$ gives

$$\begin{pmatrix} \dot{f}_A \\ \dot{f}_B \end{pmatrix} = B \begin{pmatrix} f_A \\ f_B \end{pmatrix}, \qquad B = -q^2 M_\infty H_\infty, \tag{S18}$$

where $M_\infty = M(\rho_1^\infty, \rho_2^\infty)$ and similary for H_∞. Eq. (S18) is analogous to (S7) and, for the same reasons, the eigenvalues of B are real and, if H_∞ is positive definite, negative (indicating a linearly stable fixed point).

To break the reciprocal structure, we generalise (S17) [by analogy with (S10)] as

$$\partial_t \begin{pmatrix} \rho_1 \\ \rho_2 \end{pmatrix} = \nabla \cdot \left[\mathcal{B} \begin{pmatrix} \nabla \rho_1 \\ \nabla \rho_2 \end{pmatrix} \right], \tag{S19}$$

where $\mathcal{B} = \mathcal{B}(\rho_1, \rho_2)$ is a matrix. It is not obvious in general whether such an equation can be factorised into the reciprocal form (S17). However, if the eigenvalues of $\mathcal{B}$ are complex the system can not be factorised in this way.

Physically, the most common signature of nonreciprocal effective interactions is that species 1 is attracted to species 2, while species 2 is repelled from species 1. This intuitively corresponds to $\mathcal{B}_{12}$ and $\mathcal{B}_{21}$ having opposite signs. Note however that this difference of signs may also occur in reciprocal systems,[1] so it cannot be regarded as a definite signature of nonreciprocity.

---

[1] For example, $MH = \begin{pmatrix} 8 & 3 \\ 3 & 8 \end{pmatrix} \begin{pmatrix} 4 & -1 \\ -1 & 2 \end{pmatrix} = \begin{pmatrix} 29 & -2 \\ 4 & 13 \end{pmatrix} = \mathcal{B}$.

In order to identify which systems are nonreciprocal, we write (S17) as

$$\partial_t \begin{pmatrix} \rho_1 \\ \rho_2 \end{pmatrix} = -\nabla \cdot \left[M \begin{pmatrix} \mathbf{F}_1 \\ \mathbf{F}_2 \end{pmatrix} \right], \qquad \begin{pmatrix} \mathbf{F}_1 \\ \mathbf{F}_2 \end{pmatrix} = -G \begin{pmatrix} \nabla\rho_1 \\ \nabla\rho_2 \end{pmatrix}, \tag{S20}$$

where G is a nonsymmetric matrix but M is still symmetric positive definite, and $\mathbf{F}_1, \mathbf{F}_2$ are the "forces" on species 1, 2: If $G_{12} < 0$ and $G_{21} > 0$ then species 1 feels a force towards 2, while species 2 feels a force away from 1. The systems that we consider reduce to equilibrium (reciprocal) systems in some suitable limit in which M is known. We suppose the nonreciprocity enters via the forces G so the matrix M is fixed to its equilibrium form. Then:

Definition 1 (Nonreciprocal effective interactions in cross-diffusion)**.** *Consider a (nonlinear) cross-diffusion system*

$$\partial_t \begin{pmatrix} \rho_1 \\ \rho_2 \end{pmatrix} = \nabla \cdot \left[MG \begin{pmatrix} \nabla\rho_1 \\ \nabla\rho_2 \end{pmatrix} \right] \tag{S21}$$

where M is a given 2×2 symmetric positive definite matrix. The system has nonreciprocal effective interactions if G is not symmetric.

The broken symmetry of G is analogous to taking J non-symmetric in the mechanical case of (S12), in which case Newton's third law (S9) is violated. The interesting cases tend to happen when the off-diagonal elements of G have opposite signs or G has complex eigenvalues. It may be possible to write the force as a gradient of a selfish energy $E^{(i)}$ of species i [22], for example $G_{ij}\nabla\rho_j = \nabla(\delta E^{(i)}/\delta\rho_j)$. Note that the definition (S21) does not rule out an alternative factorisation of the equation of motion as (S17) (with some different mobility M), see also [23]. However, if MG has complex eigenvalues, such a factorisation is not possible.

Example 2 (Nonreciprocal Cahn–Hilliard model [8, 24, 25])**.** In the notation of (S21), Eq. (1) of [8] has $M = \mathbf{1}$ and

$$G = H + \begin{pmatrix} 0 & \kappa - \delta \\ \kappa + \delta & 0 \end{pmatrix}, \tag{S22}$$

where H is the Hessian of $\varepsilon_{\mathrm{CH}}$, defined such that $E = \int \varepsilon_{\mathrm{CH}}(\rho_1, \rho_2)\mathrm{d}x$ is the Cahn–Hilliard energy and

$$\varepsilon_{\mathrm{CH}}(\rho_1, \rho_2) = \sum_{i=1,2} \left(\frac{\chi_i}{2}\rho_i^2 + \frac{1}{12}\rho_i^4 + \frac{\gamma_i}{2}|\nabla\rho_i|^2 \right). \tag{S23}$$

The equilibrium baseline for this model is $\delta = 0 = \kappa$. For $\delta \neq 0$, the matrix G is not symmetric and the model is nonreciprocal according to Definition 1. Further, for $|\delta| > |\kappa|$, the off-diagonal elements of G have opposite signs and, e.g., species 1 likes species 2 but species 2 dislikes species 1. When $\delta^2 > \kappa^2 + \alpha$, where α is a positive constant that depends on the self-interaction terms in E, the eigenvalues of G are complex conjugates and traveling patterns emerge [8].

Example 3 (APLG model (S1))**.** The APLG in (S1) is not a cross-diffusion system because the densities $\rho_\pm$ are not individually conserved. To identify the effective nonreciprocal interactions, we consider solutions with macroscopically smooth densities, which obey the outer equations of the matched asymptotic analysis. Specifically, writing (8) in terms of the two conserved densities $\rho_a(x,t), \rho_0(x,t)$ in the fixed reference frame, one obtains a cross-diffusion system

$$\begin{aligned} \partial_t\rho_a &= \partial_x \left[(d_s + \rho_a\mathcal{D})\partial_x\rho_a + \rho_a\mathcal{D}\partial_x\rho_0 + \frac{1}{2}\mathrm{Pe}^2(s\rho_a + d_s)\partial_x(d_s\rho_a) \right], \\ \partial_t\rho_0 &= \partial_x \left[(d_s + \rho_0\mathcal{D})\partial_x\rho_0 + \rho_0\mathcal{D}\partial_x\rho_a + \frac{1}{2}\mathrm{Pe}^2 s\rho_0\partial_x(d_s\rho_a) \right], \end{aligned} \tag{S24}$$

The corresponding equilibrium model is $\mathrm{Pe} = 0$, where (S24) is reciprocal with the mobility matrix [3, 6]

$$M = \frac{1-\rho}{\rho} \begin{pmatrix} \rho_a^2 & \rho_a\rho_0 \\ \rho_a\rho_0 & \rho_0^2 \end{pmatrix} + \frac{\rho_a\rho_0}{\rho} d_s(\rho) \begin{pmatrix} 1 & -1 \\ -1 & 1 \end{pmatrix}, \tag{S25}$$

and $E = \int \varepsilon(\rho_0, \rho_a)dx$, where

$$\varepsilon = \rho_0 \log\rho_0 + \rho_a \log\rho_a + (1-\rho)\log(1-\rho). \tag{S26}$$

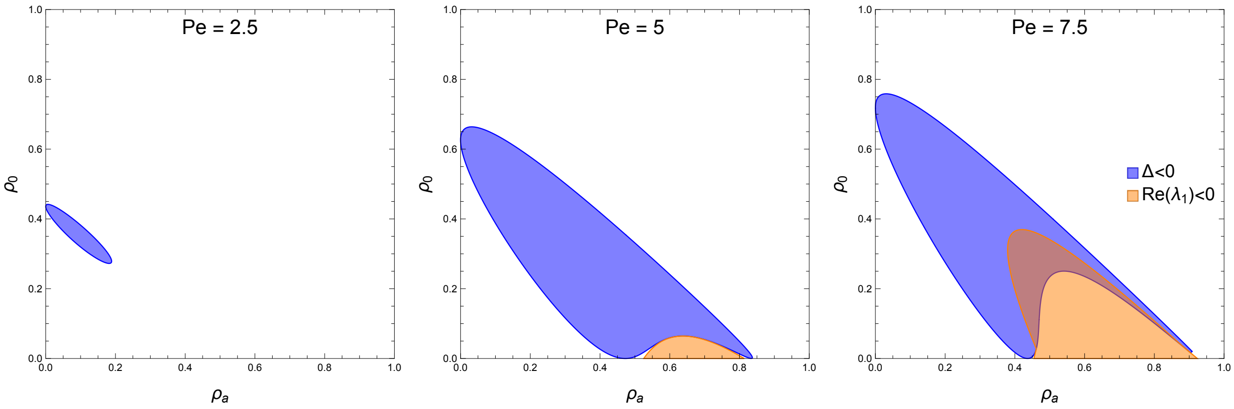

FIG. S1. **Spectrum of the cross-diffusion APLG system.** Illustrative features of the spectrum of $MG = MH + \frac{1}{2}\mathrm{Pe}^2 MG_{\mathrm{NR}}$ as defined in (S27), (S28), for various values of Pe (Pe $= 2.5, 5, 7.5$). Denoting the eigenvalues of MG by λ_1, λ_2, the blue region indicates complex eigenvalues (with discriminant $\Delta = (\lambda_1+\lambda_2)^2 - 4\lambda_1\lambda_2 < 0$) and the orange region indicates that at least one eigenvalue has negative real part ($\mathrm{Re}(\lambda_i) < 0$). Where the blue and orange regions overlap, the instability involves traveling and growing sinusoids; otherwise, the orange region corresponds to sinusoids that grow but do not travel.

Writing H for the Hessian of ε, we obtain for $\mathrm{Pe} = 0$

$$\partial_t \begin{pmatrix}\rho_a \\ \rho_0\end{pmatrix} = \partial_x \left[MH \begin{pmatrix}\partial_x \rho_a \\ \partial_x \rho_0\end{pmatrix}\right], \qquad MH = \frac{1}{\rho}\begin{pmatrix}\rho_a + d_s\rho_0 & \rho_a(1-d_s) \\ \rho_0(1-d_s) & \rho_0 + d_s\rho_a\end{pmatrix}. \tag{S27}$$

Note that, in this case, MH is not symmetric but the system is reciprocal (since H is symmetric).

Then (S24) becomes for $\mathrm{Pe} > 0$:

$$\partial_t \begin{pmatrix}\rho_a \\ \rho_0\end{pmatrix} = \partial_x \left[M(H + \tfrac{\mathrm{Pe}^2}{2} G_{\mathrm{NR}}) \begin{pmatrix}\partial_x \rho_a \\ \partial_x \rho_0\end{pmatrix}\right], \qquad MG_{\mathrm{NR}} = \begin{pmatrix}(s\rho_a + d_s)(d_s + d_s'\rho_a) & (s\rho_a + d_s)d_s'\rho_a \\ s\rho_0(d_s + d_s'\rho_a) & s\rho_0 d_s'\rho_a\end{pmatrix}, \tag{S28}$$

with M still given in (S25). Inverting M, we find that G_{NR} is not symmetric, signalling that (S24) is nonreciprocal for $\mathrm{Pe} > 0$, according to Definition 1.

Pursuing further this example by considering the eigenvalues of the matrix $MG = M(H + \frac{1}{2}\mathrm{Pe}^2 G_{\mathrm{NR}})$, we find that the spectrum is complex in general (see Fig. S1). For the region with complex eigenvalues, there is no possible factorisation of (S24) in the manifestly reciprocal form (S17). In practice, complex eigenvalues appear for $\mathrm{Pe} \gtrsim 2.5$ and complex eigenvalues with negative real part (indicating oscillatory instabilities) for $\mathrm{Pe} \gtrapprox 5$. Note the matrix MG controls the linear stability of homogeneous states but it differs from the matrix of linear stability by a sign, c.f. (S18). Hence the region where at least one eigenvalue of MG has negative real part in Fig. S1 approximates well the area under the spinodal curves in Fig. S3, see further details in Sec. D 1 below.

As a final note, we consider the outer problem (S24) in a mean-field approximation [6], such that $d_s = 1 - \rho$ and hence $\mathcal{D}(\rho) \equiv 1, s(\rho) \equiv 0$. The system still takes the form (S28) with H as above, and

$$M = (1 - \rho_a - \rho_0)\begin{pmatrix}\rho_a & 0 \\ 0 & \rho_0\end{pmatrix}, \qquad G_{\mathrm{NR}} = \begin{pmatrix}-2 + \frac{1-\rho_0}{\rho_a} & -1 \\ 0 & 0\end{pmatrix}, \tag{S29}$$

Recalling (S20), the Pe-dependent force on the active particles is

$$F_a = \frac{\mathrm{Pe}^2}{2}\left(\partial_x \rho_0 + \left[2 - \frac{1-\rho_0}{\rho_a}\right]\partial_x \rho_a\right) \tag{S30}$$

while there is no such force on the passive particles. The first term (cross-diffusion) means that active particles feel a force towards passive particles. In a reciprocal system, the passive particles would feel a corresponding attraction towards the active ones, but this is absent here, so the effective interaction is nonreciprocal. (The second term in (S30) means that active particles also attract each other if ρ_a is large enough: this is the effective attraction that drives MIPS.)

C. THE METHOD OF COEXISTING PHASES AND THE BINODAL CURVE

Equation (12) of the main text is $\partial_x g = 0$ with

$$g(\rho) = g_0(\rho) + \Lambda(\rho)(\partial_x\rho)^2 - \kappa(\rho)\partial_x^2\rho, \tag{S31}$$

where

$$g_0(\rho) = \mathrm{Pe}\Big[(1+\nu)\rho - \nu\Big]d_s(\rho) - \frac{2}{\mathrm{Pe}}\log(1-\rho), \tag{S32}$$

and

$$\Lambda(\rho) = \frac{-2d_s(\rho)}{\mathrm{Pe}(1-\rho)^2}, \qquad \kappa(\rho) = \frac{d_s(\rho)}{\mathrm{Pe}(1-\rho)}. \tag{S33}$$

We now use the method of Refs. [4, 26, 27] to derive the densities (ϕ_v, ϕ_l) of the coexisting phases. The function g is constant in space; we denote its value by $\bar{g}$. As stated in Methods, gradients of ρ vanish within the bulk of the coexisting phases so

$$g_0(\phi_v) = g_0(\phi_l) = \bar{g}. \tag{S34}$$

Next we outline the derivation of the effective free energy Φ from which ϕ_v, ϕ_l follow by the common tangent construction, see Ref. [4, 26, 27] for details. First define a (one-to-one) function $R(\rho)$ such that $\kappa R'' = -(2\Lambda + \kappa')R'$ where primes denote derivatives. Then, the effective free energy is a function $\Phi(R)$ defined (up to an additive constant) by $\Phi'(R(\rho)) = g_0(\rho)$. The definition of R is chosen such that

$$\begin{aligned}\partial_x[\kappa(\rho)R'(\rho)(\partial_x\rho)^2] &= \kappa'(\rho)R'(\rho)(\partial_x\rho)^3 + \kappa(\rho)R''(\rho)(\partial_x\rho)^3 + 2\kappa(\rho)(\partial_x^2\rho)R'(\rho)\partial_x\rho \\ &= 2[\kappa(\rho)(\partial_x^2\rho) - \Lambda(\rho)(\partial_x\rho)^2]R'(\rho)\partial_x\rho,\end{aligned} \tag{S35}$$

which will be useful below.

To see the common tangent, consider the difference in $\Phi(R(\rho))$ between two points x_v, x_l, one in the bulk of each phase. The density varies in space between the two points as $\rho = \rho(x)$ and we have

$$\begin{aligned}\Phi(R(\phi_l)) - \Phi(R(\phi_v)) &= \int_{x_v}^{x_l} \Phi'(R(\rho))R'(\rho)\partial_x\rho\, dx = \int_{x_v}^{x_l} g_0(\rho)R'(\rho)\partial_x\rho\, dx \\ &= \int_{x_v}^{x_l} g(\rho,\partial_x\rho,\partial_x^2\rho)R'(\rho)\partial_x\rho\, dx + \int_{x_v}^{x_l}[\kappa(\rho)\partial_x^2\rho - \Lambda(\rho)(\partial_x\rho)^2]R'(\rho)\partial_x\rho\,\mathrm{d}x,\end{aligned} \tag{S36}$$

where the first equality is the chain rule, the second is the definition of Φ, and the third is (S31). Using (S35), the last integrand in (S36) is a total derivative, that is

$$\int_{x_v}^{x_l}[\kappa(\rho)\partial_x^2\rho - \Lambda(\rho)(\partial_x\rho)^2]R'(\rho)\partial_x\rho\,\mathrm{d}x = \frac{1}{2}\left[\kappa(\rho)R'(\rho)\partial_x\rho\right]_{x=x_v}^{x_l} = 0. \tag{S37}$$

The last equality holds because $\partial_x\rho = 0$ in the bulk of the phases. Using this in (S36) and observing that $g = \bar{g}$ is constant in space, we find

$$\Phi(R(\phi_l)) - \Phi(R(\phi_v)) = \bar{g}\int_{x_v}^{x_l}\partial_x R(\rho)\mathrm{d}x = \bar{g}[R(\phi_l) - R(\phi_v)]. \tag{S38}$$

Finally, using (S34) and the definition of Φ we have that $\bar{g} = \Phi'(R(\phi_l)) = \Phi'(R(\phi_v))$ so introducing the shorthand notation $R_l = R(\phi_l)$ and $R_v = R(\phi_v)$, (S34) becomes $\bar{g} = \Phi'(R_l) = \Phi'(R_v)$ and (S38) yields

$$\Phi(R_l) - R_l\Phi'(R_l) = \Phi(R_v) - R_v\Phi'(R_v), \tag{S39}$$

while (S34) is

$$\Phi'(R_l) = \Phi'(R_v). \tag{S40}$$

Eqs. (S39)-(S40) are exactly the common tangent construction (convex hull) of Φ, as required. The functions Φ and R are easily determined from their definitions via numerical integration, so it only remains to solve the two simultaneous equations (S39)-(S40). Note in particular that the definition of R can be used together with (S33) to obtain

$$R'(\rho) = \frac{1}{d_s(\rho)(1-\rho)^3}. \tag{S41}$$

(The definition fixes R' up to an arbitrary multiplicative constant, set to unity here.)

D. LINEAR STABILITY OF HOMOGENEOUS SOLUTIONS AND THE SPINODAL CURVE

The homogeneous solution $(\rho, \rho_a, m) = (\phi, \phi_a, 0)$ is always a solution of Eqs. (S1). We analyze the linear stability of this solution by taking $\rho = \phi + \delta\tilde{\rho}$, $\rho_a = \phi_a + \delta\tilde{\rho}_a$, $m = 0 + \delta\tilde{m}$ with y-independent perturbations $\tilde{\rho}, \tilde{\rho}_a, \tilde{m}$. At first order in δ, we obtain

$$\partial_t\tilde{\rho} = \partial_x^2\tilde{\rho} - \mathrm{Pe}\partial_x\left[(1-\phi)\tilde{m}\right], \tag{S42}$$

$$\partial_t\tilde{\rho}_a = \partial_x\left[d_s(\phi)\partial_x\tilde{\rho}_a + \phi_a\mathcal{D}(\phi)\partial_x\tilde{\rho}\right] - \mathrm{Pe}\partial_x\left[\phi_a s(\phi)\tilde{m} + d_s(\phi)\tilde{m}\right], \tag{S43}$$

$$\partial_t\tilde{m} = \partial_x\left[d_s(\phi)\partial_x\tilde{m}\right] - \mathrm{Pe}\partial_x\left[d_s(\phi)\tilde{\rho}_a + d_s'(\phi)\phi_a\tilde{\rho}\right] - 2\tilde{m}. \tag{S44}$$

Taking a solution of the form

$$(\tilde{\rho}, \tilde{\rho}_a, \tilde{m}) = (A_1, A_2, A_3)\exp(\lambda t + iqx) \tag{S45}$$

yields

$$\begin{aligned}
\lambda A_1 &= -q^2A_1 - iq\mathrm{Pe}(1-\phi)A_3,\\
\lambda A_2 &= -q^2\phi_a\mathcal{D}(\phi)A_1 - q^2 d_s(\phi)A_2 - iq\mathrm{Pe}[\phi_a s(\phi) + d_s(\phi)]A_3,\\
\lambda A_3 &= -iq\mathrm{Pe}d_s'(\phi)\phi_a A_1 - iq\mathrm{Pe}d_s(\phi)A_2 - [q^2 d_s(\phi) + 2]A_3.
\end{aligned} \tag{S46}$$

Therefore λ is an eigenvalue of the 3×3 matrix

$$W = -\begin{pmatrix} q^2 & 0 & iq\mathrm{Pe}(1-\phi) \\ q^2\phi_a\mathcal{D}(\phi) & q^2 d_s(\phi) & iq\mathrm{Pe}[\phi_a s(\phi) + d_s(\phi)] \\ iq\mathrm{Pe}d_s'(\phi)\phi_a & iq\mathrm{Pe}d_s(\phi) & q^2 d_s(\phi) + 2 \end{pmatrix}. \tag{S47}$$

This matrix is not Hermitian, so its eigenvalues are, in general, complex. However, if the spectrum is complex, then two of the eigenvalues form a complex conjugate pair and the other remains real; this is due to symmetry under $\lambda \mapsto \bar{\lambda}$ and $(A_1, A_2, A_3) \mapsto (\bar{A}_1, \bar{A}_2, -\bar{A}_3)$ in (S46), where $\bar{A}$ denotes the complex conjugate of A.

As usual, the homogeneous state is linearly stable if all eigenvalues λ have negative real parts. (It is implicit that λ depends on q; this condition must hold for all q.) The resulting instabilities can have several types; we classify them according to the scheme of Ref. [28]. Our system has two conserved densities ρ, ρ_a and a nonconserved magnetization m. The behavior is controlled by the conserved densities, which restricts the behavior to four of the eight types considered in Ref. [28]. If the dominant eigenvalue of W is real then it is called stationary, else it is called oscillatory; if the instability is initiated by modes with $q \to 0$ then it is called large scale, else it is called small scale. The resulting types are then conserved-Turing (stationary, small scale), Cahn–Hilliard (stationary, large scale), conserved-Hopf (oscillatory, large scale), or conserved-wave (oscillatory, small scale).

Fig. S2 illustrates the range of possible behavior, showing several instabilities that occur on increasing ϕ_a at fixed total density ϕ. Row (a) shows the onset of a Cahn-Hilliard instability (stationary, large-scale), row (b) shows the onset of a conserved-Hopf instability (oscillatory, large-scale), and row (c) shows the onset of a conserved-wave instability (oscillatory, small-scale). In the left (right) column, the active volume fraction is sub(super)-critical $\phi_a < \phi_a^*$ where $\max_q \mathrm{Re}\lambda < 0$ ($\phi_a > \phi_a^*$ where $\max_q \mathrm{Re}\lambda > 0$). The central column displays the critical active volume fraction $\phi_a = \phi_a^*$ corresponding to $\max_q \mathrm{Re}\lambda/q^2 = 0$. We show below that these are the only possible scenarios because the onset of a stationary instability must occur on a large scale.

1. Spinodal curves

The main aim of this analysis is to compute spinodal curves, as shown in Fig. 1. To this end, note that any eigenvalue of W obeys the cubic equation

$$\lambda^3 - \mathrm{Tr}(W)\lambda^2 + F(W)\lambda - \det(W) = 0, \tag{S48}$$

where

$$F(W) = q^2\left\{2 + 2d_s(\phi)(1+q^2) + d_s^2(\phi)(\mathrm{Pe}^2 + q^2) + d_s'(\phi)\phi_a(1-\phi)\mathrm{Pe}^2 + d_s(\phi)\phi_a\mathrm{Pe}^2[\mathcal{D}(\phi) - 1]\right\}. \tag{S49}$$

At the boundary of linear stability, then $\mathrm{Re}(\lambda) = 0$ for at least one eigenvalue. There are two situations where this can happen

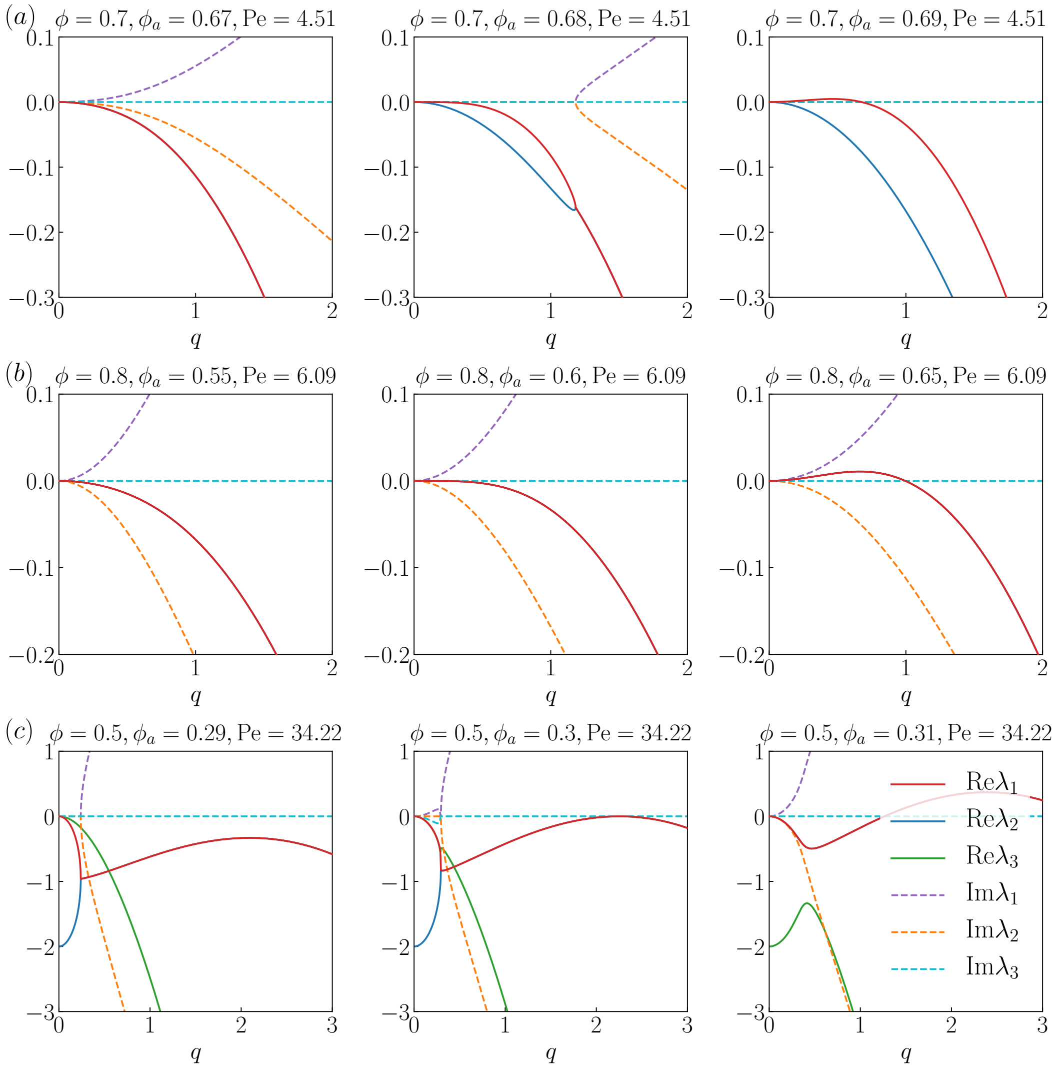

FIG. S2. **Linear stability of the APLG system.** Eigenvalues of W (S47) at the onset of instability. Active volume fraction ϕ_a increases from left to right. The critical volume fraction is displayed in the central column. (a) Cahn-Hiliard instability (stationary, large-scale). (b) Conserved-Hopf instability (oscillatory, large-scale). (c) Conserved-wave instability (oscillatory, small-scale).

(i) $\det(W) = 0$, corresponding to a vanishing eigenvalue $\lambda = 0$ (stationary instability).

(ii) the characteristic polynomial is of the form $(\lambda^2 + F(W))(\lambda - \mathrm{Tr}(W)) = 0$, with $F(W) > 0$ so that $\lambda = \pm i\sqrt{F(W)}$ is pure imaginary. This situation holds if and only if $F(W)\mathrm{Tr}(W) = \det(W) < 0$ (we have always $\mathrm{Tr}(W) < 0$ so the final inequality ensures $F(W) > 0$).

In the stationary case (i) one may solve $\det W = 0$ for ϕ_a to obtain

$$\phi_a^{\mathrm{st}}(q) = \frac{2 + d_s(\phi)\mathrm{Pe}^2 + d_s(\phi)q^2}{\mathrm{Pe}^2[d_s(\phi) - (1-\phi)d_s'(\phi)]}. \tag{S50}$$

In the oscillatory case (ii), one may similarly solve $\det(W) = F(W)\mathrm{Tr}(W)$ to obtain

$$\phi_a^{\mathrm{osc}}(q) = \frac{2\phi\left(1 + d_s(\phi)q^2\right)\left[2 + 2d_s(\phi) + d_s^2(\phi)\mathrm{Pe}^2 + \left(d_s(\phi)+1\right)^2 q^2\right]}{\mathrm{Pe}^2\left\{2d_s^3(\phi)q^2 + d_s'(\phi)(\phi-1)\phi(2+q^2) + d_s^2(\phi)[2 + (\phi-1)q^2] + d_s(\phi)(\phi-1)[2 + (1 + d_s'(\phi)\phi)q^2]\right\}}. \tag{S51}$$

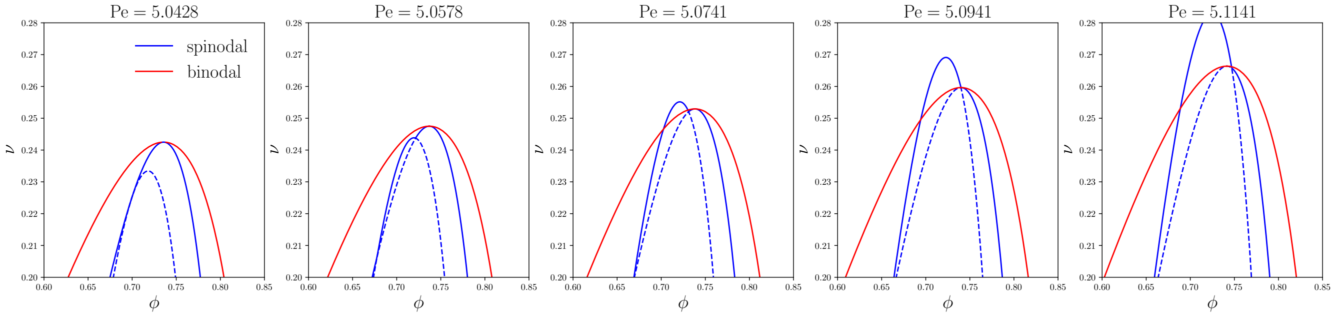

FIG. S3. **Protrusion of the spinodal beyond the binodal.** Phase diagrams spanned by ϕ and ν. The spinodal (solid blue) encloses the region of linear stability of homogeneous solutions. The dashed (solid) blue line indicates the minimum (maximum) of (S50) and (S51). The binodal (red) encloses the region of phase separation. (a-b) spinodal is contained within the binodal (c-f) spinodal protrudes through the binodal, creating instability in PS solutions.

For $\phi_a \to 0$, one sees that all eigenvalues of (S47) have negative real parts: the system is stable and $\det(\mathrm{W}) < 0$. On increasing ϕ_a at fixed ϕ, it follows that the system first becomes unstable at $\phi_a = \phi_a^*$ with

$$\phi_a^* = \inf_q \left[\min(\phi_a^{\mathrm{osc}}(q), \phi_a^{\mathrm{st}}(q)) \right]. \tag{S52}$$

[For a finite system, the trial solution (S45) is restricted to $q = 2n\pi/L$ with $n \in \mathbb{Z}$ and we should minimize over this discrete set, so ϕ_a^* depends in general on L. We consider the limit $L \to \infty$ here, so we take an infimum over $q > 0$.] Note also that, if the infimum in (S52) is achieved by $\phi_a^{\mathrm{osc}}(q)$, then it is certain that $\det(W) < 0$ at this point, as required for an oscillatory instability: this holds because $\det(W)$ only changes sign at $\phi_a = \phi_a^{\mathrm{st}}(q) > \phi_a^{\mathrm{osc}}(q)$ and $\det(W) < 0$ for $\phi_a \to 0$. We also observe from (S50) that $\inf_q \phi_a^{\mathrm{st}}(q) = \phi_a^{\mathrm{st}}(0)$, which means that if this instability is of stationary type then it is always large-scale, as already asserted above. On the other hand, the infimum of $\phi_a^{\mathrm{osc}}(q)$ may occur as $q \to 0$ (large-scale oscillatory instability) or at finite q (small-scale oscillatory instability).

Having determined ϕ_a^* in this way (and keeping fixed ϕ), the system always remains unstable for all $\phi_a > \phi_a^*$ (because $\det W > 0$ and $\mathrm{Tr}(W) < 0$). This means that exchanging passive for active particles cannot restore stability, which may be expected on physical grounds. Hence, the spinodal curve in the ϕ, ϕ_a plane is given by $\phi_a = \phi_a^*$.

For numerical calculations, it is convenient to parameterize the dependence on ϕ_a in terms of the quantity $\nu = \phi_p/(1-\phi)$ defined in Eq. (5). Then $\nu = \frac{\phi}{1-\phi}$ corresponds to a system of purely passive particles and $\nu = 0$ to purely active particles. Fig. S3 illustrates the spinodal in the (ϕ, ν)-plane. We consider a narrow range of Pe in which both oscillatory and stationary instabilities occur, and we plot separately the curves corresponding to $\phi_a = \inf_q \phi_a^{\mathrm{osc}}(q)$ and $\phi_a = \inf_q \phi_a^{\mathrm{st}}(q)$. The boundary of linear instability is given by the smaller of these ϕ_a's, which corresponds to the larger of the corresponding ν's. The change in the shape of the resulting spinodal curves illustrates the transition between the two types of phase diagrams shown in Fig. S3, as the spinodal curve starts to protrude through the binodal. [Note that, in regions where $\phi_a^{\mathrm{st}} < \phi_a^{\mathrm{osc}}$, the dashed curve $\phi_a = \phi_a^{\mathrm{osc}}$ does not indicate an eigenvalue with vanishing real part because it lies in a region where $\det(W) > 0$. However, the solid blue (spinodal) curve does always indicate such an eigenvalue.]

2. Protrusion of the spinodal through the binodal

Since the binodal curve is defined by the common tangent construction on Φ of Eqs. (S39,S40), and the function $g_0(\rho) = \Phi'(R(\rho))$ in (S32), one sees that g_0 must have two turning points between ϕ_l and ϕ_v. The equality (S50) that appears in the linear stability analysis is equivalent to $g_0' = 0$. If the spinodal instability is of stationary type, this means that points of inflection of Φ correspond to spinodal instabilities, as happens in equilibrium. The analog of the critical point in equilibrium occurs when the two turning points coalesce so that $g'' = g' = 0$ (stationary point of inflection). At this point, the binodal and spinodal curves are tangent to each other, which is again analogous to equilibrium. It corresponds to a supercritical pitchfork bifurcation. The left panel of Fig. S3 illustrates this case.

However, if the instability of the homogeneous state is of oscillatory type, the spinodal is $\phi_a = \phi_a^{\mathrm{osc}}$. This condition has no direct connection with the function g_0, so the spinodal is not determined by Φ. On increasing Pe in Fig. S3, the spinodal protrudes through the binodal for a range of densities, below the critical point. Further increasing Pe, this range extends to cover the critical point itself.

This protrusion has two effects. Firstly, where the spinodal protrudes through the low-density (vapor) branch of the binodal, the corresponding PS states also become unstable (because the large domain of the vapor phase behaves like a homogeneous state at the same density). Therefore, the steady state must be dynamic as both H and PS solutions are unstable. Secondly, when the spinodal engulfs the critical point (the maximum of the binodal in Fig. S3), the H solution becomes unstable on both sides of the bifurcation. At this point, the critical bifurcation changes from supercritical to subcritical.

As a final comment in this section, note that binodal is analytic, and has a quadratic Taylor expansion near its maximum (in the ν, ϕ plane). This maximum is a critical point, and the analytic structure is relevant for the critical behaviour of the model, whose exponents are those of the Gaussian fixed point (of the renormalisation group). This effect can be traced back to the h-dependence of the APLG rates, which acts to suppress fluctuations, and enables the exact derivation of the hydrodynamic limit.

3. Small q approximation of the spinodal

Consider (S46) in the $L \gg 1$ limit, corresponding to wavenumber $q \ll 1$. From the first two equations in (S46) we find $\lambda = O(q^2)$, $A_1, A_2 = O(1)$ and $A_3 = O(q)$. As a result, we can solve for A_3 in the third equation to obtain

$$A_3 = -\frac{1}{2} iq\mathrm{Pe}\left[d_s'(\phi)\phi_a A_1 + d_s(\phi)A_2\right] + O(q^2). \tag{S53}$$

The fact that A_3 (corresponding to the perturbation $\tilde{m}$) drops for $q \ll 1$ is consistent with our asymptotic analysis in Sec. D, where we find that the magnetization vanishes at leading order. Inserting (S53) into (S46) and rearranging the resulting 2×2 system in terms of $A_a \equiv A_2$ and $A_0 = A_1 - A_2$ we find that $\tilde{\lambda}$ for $q \ll 1$ solves

$$\widetilde{W} \begin{pmatrix} A_a \\ A_0 \end{pmatrix} = \tilde{\lambda} \begin{pmatrix} A_a \\ A_0 \end{pmatrix}, \tag{S54}$$

where $\widetilde{W} = -q^2 M(H + \frac{\mathrm{Pe}^2}{2} G_{\mathrm{NR}})$ and M, H, G_{NR} coincide with the matrices in (S27,S28) corresponding to the nonreciprocal form of the outer problem. Hence, the matrix of linear stability in the limit $q \ll 1$ coincides, up to the factor $-q^2$, with the matrix product MG when writing the outer problem as a cross-diffusion problem (Example 3).

Fig. S4 overlays the spinodal calculated according to (S50) and (S51) with the approximation for small q or outer region solution for Pe $= 7.5$. We find excellent agreement between the approximation and the full calculation, indicating that for this value of Pe, our model always goes unstable through a large-scale instability (either stationary or oscillatory). This is because, for Pe $\lessapprox 8$, $\inf_q \phi_a^{\mathrm{osc}}(q) = \phi_a^{\mathrm{osc}}(0)$.

E. NUMERICAL METHODS FOR THE HYDRODYNAMIC PDES

1. Time-dependent solutions

We use a first-order finite-volume scheme to obtain one-dimensional numerical solutions $\rho_\sigma(x,t)$ to (1) for $\sigma \in \{+1, -1, 0\}$. We first rewrite the equation in the form

$$\partial_t \rho_\sigma + \partial_x \left(M_\sigma \partial_x U_\sigma\right) + \sigma m = 0, \tag{S55}$$

where M_σ are scalar mobilities and U_σ are scalar velocities. The mobilities are defined by $M_\sigma = d_s(\rho)\rho_\sigma$, and the velocities are given by

$$U_\sigma = -\Big[\partial_x \log \rho_\sigma + \partial_x Q(\rho)\Big] + \mathrm{Pe}\Big[\sigma + \frac{m s(\rho)}{d_s(\rho)}\Big], \tag{S56}$$

where $Q : [0,1] \to \mathbb{R}$ is such that $Q'(x) = \mathcal{D}(x)/d_s(x)$. (S55) is complemented with periodic boundary conditions on $[0, L]$. We note that (S55) is not a Wasserstein gradient flow as the velocities U_σ cannot be written as derivatives of an entropy (unless Pe $= 0$).

We discretize the spatial domain $[0, L]$ into N cells of length $\Delta x = L/N$ and centre $x_i = (i + 1/2)\Delta x$ for $i = 0, \ldots, N-1$. We then approximate $\rho_\sigma(x_i, t)$ by the cell averages

$$\rho_{\sigma,i}(t) = \frac{1}{\Delta x} \int_{C_i} \rho_\sigma(x,t) \mathrm{d}x. \tag{S57}$$

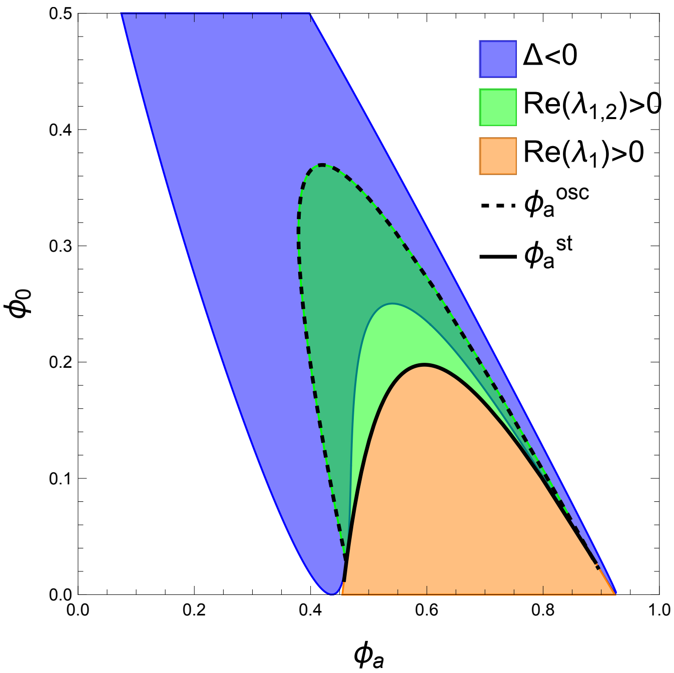

FIG. S4. **Classification of stationary and oscillatory instabilities.** Stationary and oscillatory-type instabilities in terms of ϕ_a and ϕ_0 for $\mathrm{Pe} = 7.5$. Comparison between the prediction from the 3×3 matrix W, namely $\min_q \phi_a^{\mathrm{osc}}$ and $\min_q \phi_a^{\mathrm{st}}$ using (S50) and (S51) (black solid and dashed lines) and the 2×2 matrix $\widetilde{W}$ corresponding to the $q \to 0$ limit (coloured regions). Denoting by λ_1, λ_2 the eigenvalues of $\widetilde{W}$, with $\mathrm{Re}(\lambda_1) \geq \mathrm{Re}(\lambda_2)$, the blue region indicates complex eigenvalues (with determinant $\Delta = (\lambda_1 + \lambda_2)^2 - 4\lambda_1\lambda_2 < 0$), the organge region corresponds to one eigenvalue with positive real part ($\mathrm{Re}(\lambda_1) > 0 > \mathrm{Re}(\lambda_2)$) and the green region to both eigenvalues with positive real part ($\mathrm{Re}(\lambda_{1,2}) > 0$). Where the green region overlaps with the blue region, the instability is of oscillatory type.

We use the finite-volume scheme

$$\frac{\mathrm{d}}{\mathrm{d}t}\rho_{\sigma,i} = -\frac{F_{\sigma,i+1/2} - F_{\sigma,i-1/2}}{\Delta x} - \sigma m_i. \tag{S58}$$

for $i = 0, \ldots, N-1$, with $F_{\sigma,-1/2} \equiv F_{\sigma,N-1/2}$ using periodicity. We approximate the flux F_σ at the cell interfaces by the numerical upwind flux

$$F_{\sigma,i+1/2} = d_s(\rho_{\sigma,i})\rho_{\sigma,i}(U_{\sigma,i+1/2})^+ + d_s(\rho_{\sigma,i+1})\rho_{\sigma,i+1}(U_{\sigma,i+1/2})^-, \tag{S59}$$

where $(\cdot)^+ = \max(\cdot, 0)$ and $(\cdot)^- = \min(\cdot, 0)$ and $\rho_{\sigma,N} \equiv \rho_{\sigma,0}$. The velocities U_σ are approximated by centered differences

$$U_{\sigma,i+1/2} = -\left[\frac{\log \rho_{\sigma,i+1} - \log \rho_{\sigma,i}}{\Delta x} + \frac{Q(\rho_{i+1,j}) - Q(\rho_{\sigma,i})}{\Delta x}\right] + \mathrm{Pe}\left[\sigma + \frac{1}{2}\left(\frac{m_{i+1}s(\rho_{i+1})}{d_s(\rho_{i+1})} + \frac{m_i s(\rho_i)}{d_s(\rho_i)}\right)\right]. \tag{S60}$$

Finally, the resulting system of ODEs (S58) for $\rho_{\sigma,i}(t)$ is solved by the forward Euler method with an adaptive time stepping condition satisfying

$$\Delta t = \min\left\{10^{-5}, \Delta x/(6a)\right\} \tag{S61}$$

with $a = \max_{\sigma,i}\{|U_{\sigma,i}|\}$. In [29], a CFL condition of the form (S61) is shown to result in a positivity-preserving numerical scheme. In contrast to our model, their scheme is second-order in space, using a linear density reconstruction at the interfaces that preserve positivity. Here, we follow instead [4, 30] and use the values at the center of the cells. In our numerical tests, we observe (S61) to be sufficient to preserve positivity.

We initiate the scheme with a perturbation around the homogeneous state, $\rho_\sigma(x,0) = \phi_\sigma + \delta\tilde{\rho}_\sigma(x)$ with $\phi_\pm = \phi_a/2$ and $\phi_0 = \phi_p$. We normalise the perturbation so that $\|(\tilde{\rho}_+, \tilde{\rho}_0, \tilde{\rho}_-)\|_2 = 1$, where $\|u\|_2 = \left(\int_0^L |u|^2 \mathrm{d}x\right)^{1/2}$ is the L_2 norm. For a uniformly random perturbation, we define

$$\tilde{\rho}_\sigma^{\mathrm{rand}}(x) \sim \mathrm{Unif}[-1, 1]. \tag{S62}$$

We also use the eigenfunctions from linear stability analysis to generate perturbations. In particular, we solve (S46) for $q = 2\pi/L$ and select the solution corresponding to the eigenvalue, λ, with the largest real part and nonnegative imaginary part. We define left and right traveling perturbations,

$$\tilde{\rho}_\sigma^{\mathrm{L}}(x) = \mathrm{Re}[A_\sigma \exp(iqx)], \quad \tilde{\rho}_\sigma^{\mathrm{R}}(x) = \mathrm{Re}[A_\sigma \exp(-iqx)], \tag{S63}$$

where $A_\pm = (A_2 \pm A_3)/2$, $A_0 = A_1 - A_2$. (Both perturbations will be stationary when $\mathrm{Im}\lambda = 0$.) In Figs. 2, 3 we set $\tilde{\rho}_\sigma \propto \tilde{\rho}_\sigma^{\mathrm{rand}}$ and $\delta = 0.1$ to mimic the random initial condition of the particle simulation. In Fig. 4 we set $\tilde{\rho}_\sigma \propto \tilde{\rho}_\sigma^{\mathrm{L}} + \tilde{\rho}_\sigma^{\mathrm{R}} + \tilde{\rho}_\sigma^{\mathrm{rand}}$ and $\delta = 0.1$. The left and right traveling perturbations, $\tilde{\rho}_\sigma^{\mathrm{L}} + \tilde{\rho}_\sigma^{\mathrm{R}}$, seeds the growth of counterpropagating interfaces, but the random perturbation, $\rho_\sigma^{\mathrm{rand}}$, allows asymmetry to grow. Eventually, the solution reaches a steady, left-traveling TP state. In Fig. 7 (a) we set $\tilde{\rho}_\sigma \propto \tilde{\rho}_\sigma^{\mathrm{L}} + \tilde{\rho}_\sigma^{\mathrm{R}}$ and $\delta = 0.1$. This initial condition ensures that any left and right traveling interfaces will be balanced; therefore, the steady cannot be TP. On the other hand, in Fig. 7 (b) we set $\tilde{\rho}_\sigma \propto \tilde{\rho}_\sigma^{\mathrm{L}}$ and $\delta = 0.1$. When λ is complex, this encourages left-traveling TP steady states.

2. Traveling solutions in the finite system

We seek one-dimensional traveling solutions $\varrho_\sigma(z)$ to (7) with periodic boundary conditions, where $z = x - ct/L \in [-L/2, L/2]$. Integrating (7) gives

$$-(c/L)\varrho_\sigma + M_\sigma \partial_z U_\sigma + \sigma \int_{-L/2}^{z} \mathrm{m}(y)\mathrm{d}y = A_\sigma, \tag{S64}$$

where A_σ are integration constants and the mobilities M_σ and velocities U_σ are as given in Subsec. E 1 but replacing ρ_σ by their traveling frame counterparts ϱ_σ. Additionally, we have the mass constraints

$$\frac{1}{L}\int_{-L/2}^{L/2} \varrho_\sigma \mathrm{d}z = \phi_\sigma, \tag{S65}$$

with $\phi_\pm = \phi_a/2$ and $\phi_0 = \phi_p$. [Steady-state solutions must have $\int \mathrm{m}\mathrm{d}z = 0$, as seen by integrating (7).]

We use the same finite-volume spatial discretization as in Subsec. E 1, resulting in the discretized equations

$$-\frac{c}{2L}\left(\varrho_{\sigma,i} + \varrho_{\sigma,i+1}\right) + F_{\sigma,i+1/2} + \sigma \Delta z \sum_{j=0}^{i} \mathrm{m}_j = A_\sigma, \tag{S66}$$

for $i = 0, \dots N-1$, where $F_{\sigma,i+1/2}$ is defined in (S59) (but in terms of the travelling frame variables), together with mass constraints

$$\Delta z \sum_{i=0}^{N-1} \varrho_{\sigma,i} = L\phi_\sigma, \tag{S67}$$

So far, we have $3N$ equations ($3(N-1)$ in (S66) and three in (S67)) for $3N+1$ unknowns. The remaining degree of freedom is removed by noting the problem has translational symmetry; hence, we set $\varrho_{0,0} = \phi_p$ without loss of generality.

The nonlinear system of $3N$ equations (S66)-(S67) is then solved numerically using `NonlinearSolve()` from the `Julia NonlinearSolve.jl` package [31], with parameters `reltol=1e-8`, `abstol=1e-8` and `maxiters=20`. The solver first tries less robust Quasi-Newton methods for more performance and then tries more robust techniques if the faster ones fail. As such, it requires a close enough initial guess for convergence. The first time, we initialize the iterative solver with the long-time ($T = 1000$) solution of the time-dependent problem (Subsec. E 1) with parameters $\phi = 0.67, \phi_a = 0.37$,$\mathrm{Pe} = 7.5$, $L = 25$,$N = 500$. Once we have found a traveling solution to (S66,S67), we use it as an initial condition for the problem with slightly altered parameters: $\phi_a' = \phi_a \pm 0.01$, $\phi_p' = \phi_p \pm 0.01$, as well as larger domains, e.g., $L' = 2L$ or $N' = 2N$.

3. Traveling solutions in large systems

We seek solutions $\varrho_\sigma(z)$ to (8) for $z \in [-L/2, L/2]$ with periodic boundary conditions with up to two interfaces (inner regions) at $z = 0$ and $z = \delta < L/2$. In what follows, it is convenient to consider the domain $z \in [0, L]$ instead so that one of the interfaces (if any) is at the interval ends.

We define three outer problems depending on the number of interfaces. Each of them takes different input parameters:

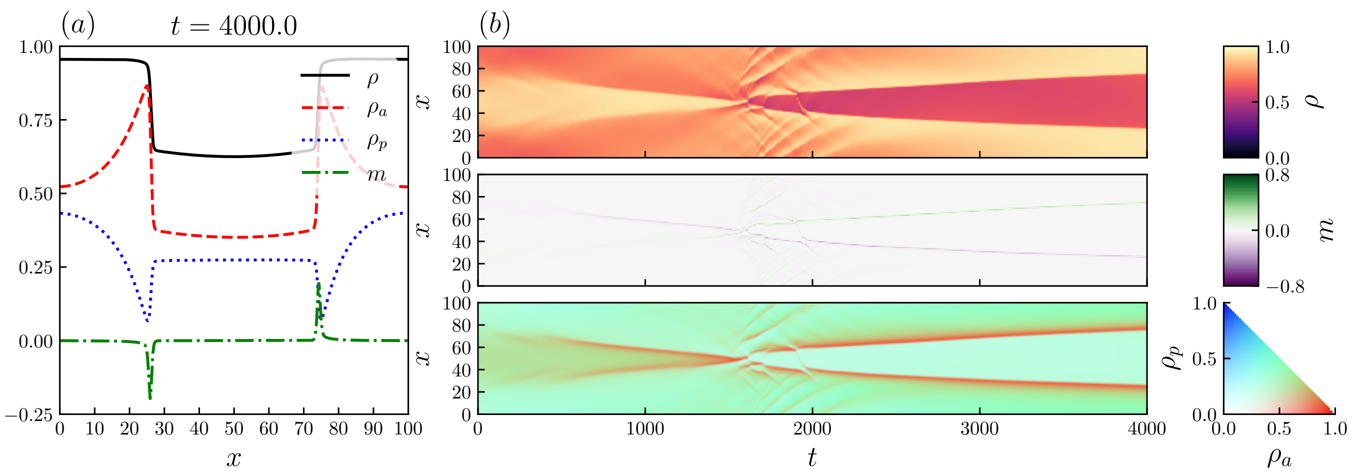

FIG. S5. **Scattering in a counter-propagating solution.** CP solution to (1). The initial condition is the homogeneous state ($\rho_\pm = \phi_a/2, \rho_0 = \phi_p$) perturbed by a uniform random perturbation (see Sec. E 1 for details). (a) Density profile at $t = 4000$. (b) Kymographs showing the spatiotemporal dynamics. Parameters: $\mathrm{Pe} = 7.5$, $L = 100.0$, $\phi_a = 0.5$, $\phi_p = 0.3$, $\Delta x = 0.05$.

- The no interface problem (`outer0`) takes the total volume fractions ϕ, ϕ_a.
- The one interface problem (`outer1`) takes the total volume fraction ϕ and the tie-line parameter ν.
- The two interfaces problem (`outer2`) takes one tie-line parameter ν and the separation between interfaces δ.

To solve the `outer0` problem, we write (8) in terms of ϱ and ϱ_a and integrate, leading to

$$\begin{aligned} -(c/L)\varrho + F &= A_1, & F &= -\varrho' - \tfrac{\mathrm{Pe}^2}{2}(1-\varrho)\partial_z[d_s(\varrho)\varrho_a] \\ -(c/L)\varrho_a + F_a &= A_2, & F_a &= -d_s(\varrho)\varrho_a' - \varrho_a \mathcal{D}(\varrho)\varrho' - \tfrac{\mathrm{Pe}^2}{2}\left[\varrho_a s(\varrho) + d_s(\varrho)\right]\partial_z[d_s(\varrho)\varrho_a], \end{aligned} \tag{S68}$$

where A_1, A_2 are constants of integration (note that we only have two equations since $\mathrm{m} = 0$ at leading order). Additionally, we have the mass constraints

$$\frac{1}{L}\int_0^L \varrho \mathrm{d}z = \phi, \qquad \frac{1}{L}\int_0^L \varrho_a \mathrm{d}z = \phi_a, \tag{S69}$$

We discretize the domain $[0, L]$ into N intervals of equal length $\Delta z = L/N$, and solve for ϱ_i and $\varrho_{a,i}$ at grid points $z_i = i\Delta z$ for $i = 0, \dots, N-1$. The discretized equations of (S68) are, for $i = 0, \dots, N-1$,

$$-\frac{c}{2L}(\varrho_i + \varrho_{i+1}) + F_{i+1/2} = A_1, \qquad -\frac{c}{2L}(\varrho_{a,i} + \varrho_{a,i+1}) + F_{a,i+1/2} = A_2, \tag{S70}$$

with the fluxes at the half-points approximated by centered differences, e.g.,

$$F_{i+1/2} = -\frac{1}{\Delta z}(\varrho_{i+1} - \varrho_i) - \frac{\mathrm{Pe}^2}{4\Delta z}(2 - \varrho_i - \varrho_{i+1})\left[d_s(\varrho_{i+1})\varrho_{a,i+1} - d_s(\varrho_i)\varrho_{a,i}\right],$$

with $\varrho_N = \varrho_0$ and $\varrho_{a,N} = \varrho_{a,0}$ using periodicity. The mass constraints (S69) become

$$\Delta z \sum_{i=0}^{N-1} \varrho_i = L\phi, \qquad \Delta z \sum_{i=0}^{N-1} \varrho_{a,i} = L\phi_a. \tag{S71}$$

This yields a set of $2N+3$ unknowns ($\varrho_i, \varrho_{a,i}, c, A_1, A_2$) and $2N+2$ equations [Eqs. (S70) and (S71)]. As in Subsec. E 2, the additional constraint comes from noting that the problem has translational symmetry; we fix $\varrho_0 = \phi$.

For the `outer1` problem with parameters ϕ and ν, we first solve the coexisting phases problem (Sec. C) given ν. This results in vapor and liquid phases $\phi_v(\nu)$ and $\phi_l(\nu)$, respectively, as well as individual components $(\varrho_\sigma)_v$ and $(\varrho_\sigma)_l$ in each phase. They satisfy $(\varrho_\sigma)_v \equiv (\varrho_+, \varrho_-, \varrho_0)_v = (\frac{1}{2}\phi_{a,g}, \frac{1}{2}\phi_{a,g}, \phi_{p,g})$ and similarly for $(\varrho_\sigma)_l$. If $\phi_v(\nu) = \phi_l(\nu)$,

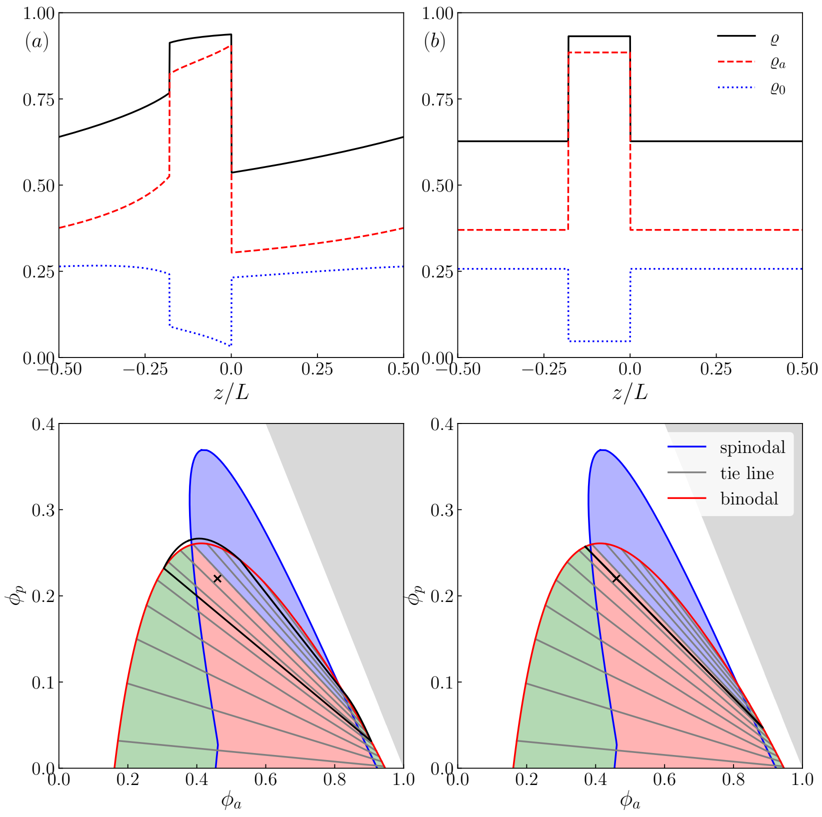

FIG. S6. **Multi-stability of solutions.** Two solutions, both obtained from Eq. (8) with the same parameters. (a) T solution. (b) PS solution. The top panels display the solution profile as a function of z/L. The bottom panels display the profile (solid black) overlayed on phase diagrams spanned by ϕ_a and ϕ_p. The Spinodal (Blue) encloses the region of linear stability of homogeneous solutions, and the Binodal (Red) encloses the region of Phase separation. Black crosses mark the volume fraction. Parameters: Pe $= 7.5$, $\phi_a = 0.4608$, $\phi_p = 0.2201$, $N = 1024$

it means there is no inner region and `outer1` has no solution (and we revert to `outer0`). Next, we solve the outer problem (S68) with Dirichlet boundary conditions $\varrho(0) = \phi_v$, $\varrho(L) = \phi_l$, $\varrho_a(0) = \phi_{a,g}$ and $\varrho_a(L) = \phi_{a,l}$. In contrast to `outer0`, we now have $2(N-1)+3$ unknowns ($\varrho_i, \varrho_{a,i}$ for $i = 1, \ldots, N-1$ and c, A_1, A_2) and $2N+1$ equations [Eqs. (S70) for $i = 0, \ldots, N-1$ and one mass constraint (S71)]. The actives volume fraction ϕ_a is found a posteriori as $\phi_a = (1/N)\sum_{i=0}^{N-1} \varrho_{a,i}$.

To obtain a solution to the `outer2` problem with two interfaces at a distance δ and the first one with a tie-line parameter ν, the first step is as in `outer1`: we solve the coexisting phases problem with ν and this gives us Dirichlet boundary conditions at $z = 0$ and $z = L$ (as before, if $\phi_v(\nu) = \phi_l(\nu)$ there is no interface and we revert to `outer1`). We take the separation between interfaces δ to be $\delta = (j + 1/2)\Delta z$ for some j, that is, the second interface lies at the half-grid point $z_{j+1/2}$. To force a second interface between the grid points z_j and z_{j+1}, the two equations (S70) corresponding to this half-grid point are replaced by three binodal equations (Sec. C)

$$\Phi(R_j) - R_j\Phi'(R_j) = \Phi(R_{j+1}) - R_{j+1}\Phi'(R_{j+1}), \qquad \Phi'(R_j) = \Phi'(R_{j+1}), \qquad \nu_j = \nu_{j+1}, \tag{S72}$$

where we introduced the shorthand $\nu_j = \frac{\varrho_j - \varrho_{a,j}}{1-\varrho_j}$ and $R_j = R(\varrho_j, \nu_j)$. Next, we solve the modified outer problem Eqs. (S70)-(S72) with the Dirichlet boundary conditions coming from the first interface and one additional constraint coming from the second interface – this explains why, in this case, the total volume fraction ϕ is not an input parameter but obtained a posteriori together with ϕ_a.

The nonlinear systems `outer0`,`outer1`,`outer2` are again solved numerically using `NonlinearSolve()` with paramters `reltol=1e-8`, `abstol=1e-8` and `maxiters=20`. We first initialise `outer0` using the finite domain solution (Subsec. E 2) with $\phi = 0.80, \phi_a = 0.45$, Pe $= 7.5$, $L = 100$, $N = 3200$. Once an initial solution to `outer0` has been found, we initialize the iterative solver using an existing solution with similar parameters: $\phi'_a = \phi_a \pm 0.01$, $\phi'_p = \phi_p \pm 0.01$. We first initialise `outer1` using the finite domain solution (Subsec. E 2) with $\phi = 0.8, \phi_a = 0.3$,Pe $= 7.5$,$N = 3200$. Once an initial solution to `outer0` has been found, we initialize the iterative solver using an existing solution

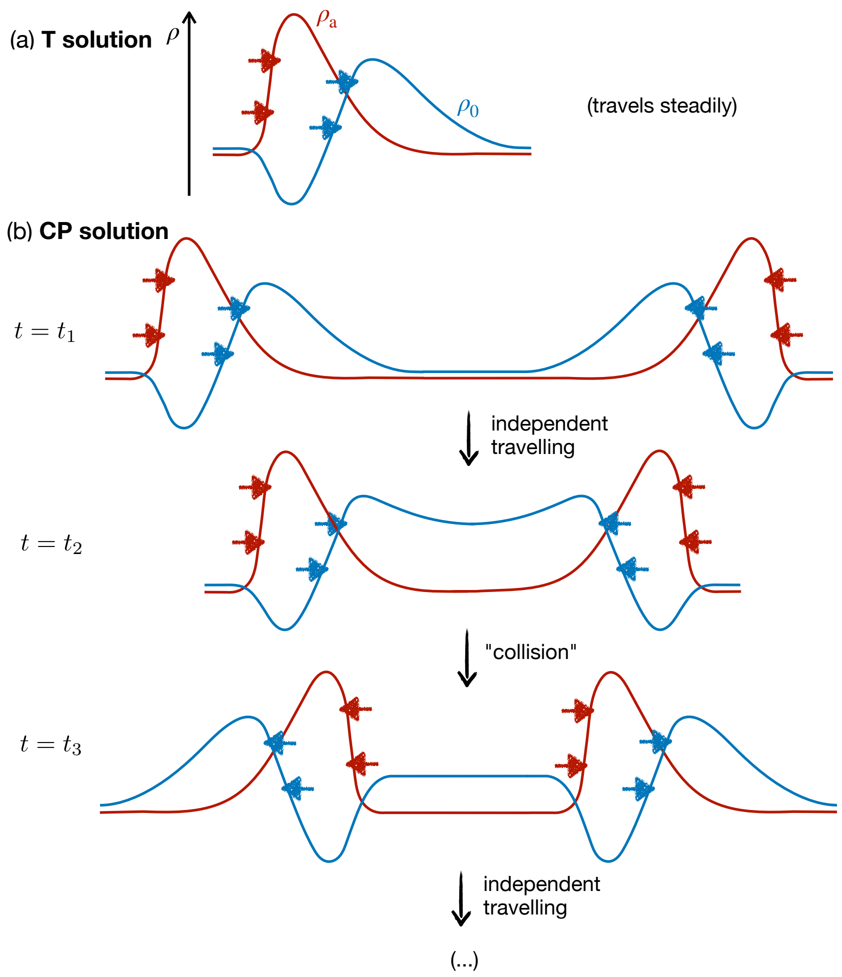

FIG. S7. **Illustration of T and CP solutions.** (a) Sketch of T solution with positive velocity, showing density of active and passive particles as a function of position (see Fig. 4 of main text for a similar solution with negative velocity). The red arrows indicate the self-propulsive force acting on the active particles. The blue arrows indicate forces applied by the active particles on the passive ones (via the cross-diffusion terms in the equation of motion). This generates the positive propagation. (b) Sketch of a CP solution at three different times in a large system (see Fig. 3 of main text for a similar solution in a smaller system). Initially the CP solution is well-approximated by a combination of two T solutions with opposite velocities of equal magnitude. The two propagating clusters approach each other and eventually collide. After the collision process one again sees a combination of two T solutions which travel away from each other. The cycle will eventually repeat due to periodic boundaries.

with similar parameters: $\phi' = \phi \pm 0.01$, $\nu' = \nu \pm 0.01$. We first initialise `outer2` using the `outer1` solution with $\phi = 0.65, \nu = 0.36$,$\mathrm{Pe} = 7.5$,$N = 1024$, and place the new interface at $j = 299$. Once an initial solution to `outer2` has been found, we initialize the iterative solver using an existing solution with similar parameters: $\nu' = \nu \pm 0.01$, $j' = j \pm 8$.

F. CP AND T SOLUTIONS

Fig. S5 illustrates a CP solution in a large domain, showing the emergence of narrow interfaces as in T states. Fig. S6 shows an example state point where both T and PS solutions can exist – this is multistability, as discussed in Sec. E of main text.

To further clarify the relationship between CP and T solutions, we sketch in Fig. S7 the qualitative behavior that we expect in large systems. The T solution involves a localized set of active particles that "push" a set of passive ones through the system (Fig. S7(a)). Red arrows indicate the positive self-propulsive forces that act in the (inner) region where ρ_a rises steeply; m is positive here and the relevant terms in the equation of motion are those proportional

to Pe. Blue arrows indicate that the passive particles are pushed by the active ones, primarily due to cross-diffusion terms in the equation of motion.

The CP solution consists of two localized sets of active particles moving in opposite directions, resembling a combination of two T solutions traveling in opposite directions (Fig. S7(b)). There are two associated inner regions where these particles propel themselves (red arrows), and the active particles push the passive ones as before. When the two traveling objects approach each other, they collide. The collision process is complex, with the clusters departing the collision at high speeds. (Note: we depict the traveling objects as localized for illustrative purposes, but the actual densities are functions of x/L in the outer region.)

An essential feature of a stable CP solution is that both traveling objects survive the collision. In contrast, while the early-time behavior shown in Fig. 4 resembles a CP solution, after three collisions, it becomes evident that the two traveling objects have unequal masses, resulting in only one object surviving after the fourth collision. The outcome for large times corresponds to a T solution.

---

[1] C. Erignoux, Hydrodynamic limit for an active exclusion process, Mémoires de la Société Mathématique de France **169**, 1 (2021).

[2] C. Kipnis and C. Landim, *Scaling Limits of Interacting Particle Systems* (Springer Berlin, Heidelberg, 1998).

[3] J. Quastel, Diffusion of color in the simple exclusion process, Commun. Pure Appl. Math. **45**, 623 (1992).

[4] J. Mason, C. Erignoux, R. L. Jack, and M. Bruna, Exact hydrodynamics and onset of phase separation for an active exclusion process, Proc. R. Soc. A Math. Phys. Eng. Sci. **479**, 20230524 (2023).

[5] C. Arita, P. Krapivsky, and K. Mallick, Variational calculation of transport coefficients in diffusive lattice gases, Physical Review E **95**, 032121 (2017).

[6] J. Mason, R. L. Jack, and M. Bruna, Macroscopic behaviour in a two-species exclusion process via the method of matched asymptotics, J. Stat. Phys. **190**, 47 (2023).

[7] C. Landim, S. Olla, and S. Varadhan, Symmetric simple exclusion process: Regularity of the self-diffusion coefficient, Communications in Mathematical Physics **224**, 307 (2001).

[8] Z. You, A. Baskaran, and M. C. Marchetti, Nonreciprocity as a generic route to traveling states, Proc. Natl. Acad. Sci. **117**, 19767 (2020).

[9] J. Stenhammar, R. Wittkowski, D. Marenduzzo, and M. E. Cates, Activity-induced phase separation and self-assembly in mixtures of active and passive particles, Physical Review Letters **114**, 018301 (2015).

[10] A. Wysocki, R. G. Winkler, and G. Gompper, Propagating interfaces in mixtures of active and passive Brownian particles, New J. Phys. **18**, 123030 (2016).

[11] D. Martin, D. Seara, Y. Avni, M. Fruchart, and V. Vitelli, The transition to collective motion in nonreciprocal active matter: coarse graining agent-based models into fluctuating hydrodynamics, arXiv preprint arXiv:2307.08251.

[12] T. Agranov, R. L. Jack, M. E. Cates, and Étienne Fodor, Thermodynamically consistent flocking: from discontinuous to continuous transitions, New Journal of Physics **26**, 063006 (2024).

[13] M. Kourbane-Houssene, C. Erignoux, T. Bodineau, and J. Tailleur, Exact hydrodynamic description of active lattice gases, Physical Review Letters **120**, 268003 (2018).

[14] T. Frohoff-Hülsmann, J. Wrembel, and U. Thiele, Suppression of coarsening and emergence of oscillatory behavior in a Cahn-Hilliard model with nonvariational coupling, Phys. Rev. E **103**, 042602 (2021).

[15] T. Frohoff-Hülsmann, U. Thiele, and L. M. Pismen, Non-reciprocity induces resonances in a two-field Cahn–Hilliard model, Philos. Trans. R. Soc. A Math. Phys. Eng. Sci. **381**, 20220087 (2023).

[16] T. Frohoff-Hülsmann and U. Thiele, Nonreciprocal Cahn-Hilliard model emerges as a universal amplitude equation, Phys. Rev. Lett. **131**, 107201 (2023).

[17] L. Yao and R. L. Jack, Interfacial and density fluctuations in a lattice model of motility-induced phase separation, arXiv:2412.04450.

[18] M. Fruchart, R. Hanai, P. B. Littlewood, and V. Vitelli, Non-reciprocal phase transitions, Nature **592**, 363 (2021).

[19] J. D. Murray, *Mathematical biology: I. An introduction*, 3rd ed. (Springer New York, NY, 2007).

[20] L. Onsager, Reciprocal relations in irreversible processes. II., Phys. Rev. **38**, 2265 (1931).

[21] A. Mielke, D. R. M. Renger, and M. A. Peletier, A generalization of onsager's reciprocity relations to gradient flows with nonlinear mobility, Journal of Non-Equilibrium Thermodynamics **41**, 141 (2016).

[22] Y. Avni, M. Fruchart, D. Martin, D. Seara, and V. Vitelli, The non-reciprocal ising model, arXiv preprint arXiv:2311.05471 (2023).

[23] P. Degond, S. Génieys, and A. Jüngel, Symmetrization and entropy inequality for general diffusion equations, Comptes Rendus de l'Académie des Sciences - Series I - Mathematics **325**, 963 (1997).

[24] R. Wittkowski, J. Stenhammar, and M. E. Cates, Nonequilibrium dynamics of mixtures of active and passive colloidal particles, New J. Phys. **19**, 105003 (2017).

[25] A. Dinelli, J. O'Byrne, A. Curatolo, Y. Zhao, P. Sollich, and J. Tailleur, Non-reciprocity across scales in active mixtures, Nat. Commun. **14**, 7035 (2023).

[26] A. P. Solon, J. Stenhammar, M. E. Cates, Y. Kafri, and J. Tailleur, Generalized thermodynamics of motility-induced phase

separation: Phase equilibria, Laplace pressure, and change of ensembles, New J. Phys. **20**, 075001 (2018).
[27] A. P. Solon, J. Stenhammar, M. E. Cates, Y. Kafri, and J. Tailleur, Generalized thermodynamics of phase equilibria in scalar active matter, Phys. Rev. E **97**, 020602 (2018).
[28] T. Frohoff-Hülsmann and U. Thiele, Nonreciprocal Cahn-Hilliard model emerges as a universal amplitude equation, Physical Review Letters **131**, 107201 (2023).
[29] N. Kruk, J. A. Carrillo, and H. Koeppl, A finite volume method for continuum limit equations of nonlocally interacting active chiral particles, J. Comput. Phys. **440**, 110275 (2021).
[30] M. Bruna, M. Burger, A. Esposito, and S. M. Schulz, Phase separation in systems of interacting active brownian particles, SIAM J. Appl. Math. **82**, 1635 (2022).
[31] A. Pal, F. Holtorf, A. Larsson, T. Loman, F. Schaefer, Q. Qu, A. Edelman, C. Rackauckas, *et al.*, Nonlinearsolve. jl: High-performance and robust solvers for systems of nonlinear equations in Julia, Preprint arXiv:2403.16341 (2024).
